# Informant-reported cognitive symptoms that predict amnestic mild cognitive impairment

**DOI:** 10.1186/1471-2318-12-3

**Published:** 2012-02-03

**Authors:** Michael Malek-Ahmadi, Kathryn Davis, Christine M Belden, Sandra Jacobson, Marwan N Sabbagh

**Affiliations:** 1 The Cleo Roberts Center for Clinical Research, Banner Sun Health Research Institute, Sun City, AZ, 85351, USA

## Abstract

**Background:**

Differentiating amnestic mild cognitive impairment (aMCI) from normal cognition is difficult in clinical settings. Self-reported and informant-reported memory complaints occur often in both clinical groups, which then necessitates the use of a comprehensive neuropsychological examination to make a differential diagnosis. However, the ability to identify cognitive symptoms that are predictive of aMCI through informant-based information may provide some clinical utility in accurately identifying individuals who are at risk for developing Alzheimer's disease (AD).

**Methods:**

The current study utilized a case-control design using data from an ongoing validation study of the Alzheimer's Questionnaire (AQ), an informant-based dementia assessment. Data from 51 cognitively normal (CN) individuals participating in a brain donation program and 47 aMCI individuals seen in a neurology practice at the same institute were analyzed to determine which AQ items differentiated aMCI from CN individuals.

**Results:**

Forward stepwise multiple logistic regression analysis which controlled for age and education showed that 4 AQ items were strong indicators of aMCI which included: repetition of statements and/or questions [OR 13.20 (3.02, 57.66)]; trouble knowing the day, date, month, year, and time [OR 17.97 (2.63, 122.77)]; difficulty managing finances [OR 11.60 (2.10, 63.99)]; and decreased sense of direction [OR 5.84 (1.09, 31.30)].

**Conclusions:**

Overall, these data indicate that certain informant-reported cognitive symptoms may help clinicians differentiate individuals with aMCI from those with normal cognition. Items pertaining to repetition of statements, orientation, ability to manage finances, and visuospatial disorientation had high discriminatory power.

## Background

The process of differentiating age-associated memory decline from those who might have a clinically significant disorder of memory and cognition is difficult. In particular, distinguishing individuals with amnestic mild cognitive impairment (aMCI) from those who are cognitively normal (CN) is challenging, as memory and cognitive complaints are often reported in both groups from both the patient and informants [1]. Given that the current diagnostic criteria for aMCI include subjective (patient and/or family report of decline) and objective (neuropsychological testing) evidence of memory decline, a clinician's initial impression from a relatively short office visit may not allow for an accurate assessment [2].

Amnestic MCI was first characterized as a syndrome consisting of memory performance at or below 1.5 standard deviations (SD) on age- and education-adjusted normative values on a verbal memory test along with subjective memory complaints, preserved global cognition, and preserved activities of daily living [3]. The diagnostic criteria for MCI have since been refined to differentiate between amnestic and non-amnestic forms, with the latter showing performance at or below 1.5 SD on a test or test(s) in one or more domains other than memory. Both amnestic and non-amnestic MCI can be further classified as single or multiple domain MCI depending upon the number of cognitive domains that show test performance(s) at or below 1.5 SD [4].

Several studies have investigated the clinical course and presentation of individuals who have self- and informant-reported memory complaints [5-8]. Some evidence suggests that individuals who are cognitively normal and have subjective memory complaints demonstrate MRI findings that are similar to those of aMCI individuals [9]. Other studies have demonstrated that an informant's report of an individual's cognitive status is valid and highly accurate in the very early stages of AD [6]. Although the diagnostic criteria for aMCI do not include functional impairment, previous studies have found that aMCI patients may have difficulty with higher level daily activities, such as balancing a checkbook, and may show mild, but not significant, difficulty in daily functioning [1,10].

Utilizing additional information with added discriminatory power can aid in identifying individuals at risk for developing Alzheimer's disease (AD), a task of greater interest now, with emerging early AD treatments [10,11]. To accomplish this, identifying certain cognitive symptoms that may yield greater diagnostic accuracy than subjective memory complaints alone is necessary. A recent pilot study found that the Alzheimer's Questionnaire (AQ), an informant-based questionnaire designed for use in primary care settings, has both high sensitivity [87.00 (77.00 - 94.00)] and specificity [94.00 (84.00, 99.00)] for aMCI [12].

The intent of this study is to determine which AQ items are predictive of aMCI. By identifying cognitive symptoms beyond subjective memory complaints, individuals at risk for developing AD may be identified more quickly so that further diagnostic testing and subsequent treatment may be initiated sooner in the disease process.

## Method

### Study Sample

Data from 98 individuals (47 aMCI, 51 CN) were taken from an ongoing validation study of the AQ. Both aMCI and CN individuals were drawn from the same geographic population (Sun City, AZ). A case-control design was used for this study as the aMCI participants were drawn from the practice of a neurologist specializing in dementia and memory disorders. The clinician's diagnosis was used as the gold standard for aMCI participants, based on cognitive and medical history, informant interview, and neuropsychological testing utilizing Petersen criteria [3]. Individuals whose performance was 1.5 standard deviations (SD) below age- and education-corrected means on a delayed recall measure of verbal memory were classified as aMCI. Individuals with both single and multiple domain aMCI were included in the analysis. Multiple domain aMCI cases were classified as those with memory performance 1.5 SD below age- and education-corrected means with performance in another cognitive domain (e.g., executive functions) also falling 1.5 SD below age- and education-corrected means.

CN participants were drawn from a brain and body donation program in which informants were given the AQ as part of the participants' annual assessment. Both aMCI and CN participants were recruited consecutively. CN participants were defined as having no demonstrable cognitively-based limitations of activities of daily living through an informant interview by a physician. In addition, all CN participants scored above 1.5 SD on age- and education-corrected means on a battery of neuropsychological tests and received global CDR rating of 0 [13]. Consensus diagnosis with a neurologist, geriatric psychiatrist, and neuropsychologist was used as the gold standard in determining CN status. The AQ was not utilized in the differential diagnosis for aMCI individuals and was not utilized in the consensus diagnosis for CN individuals. Interviews with the participant and informant and review of medical records were used to exclude those with symptomatic or severe brain-related neurological or psychiatric illness. Excluded conditions included mental retardation, epilepsy, cerebral infarction or hemorrhage, multiple sclerosis, brain tumor, major depressive disorder (unipolar or bipolar), schizophrenia, traumatic brain injury, and substance abuse. Collateral informants provided additional information on cognitive and functional changes.

IRB approval was waived by the Sun Health IRB as the study fell under their categorization of research involving the use of educational tests (cognitive, diagnostic, aptitude, achievement), survey procedures, interview procedures or observation of public behavior which is not subject to review and does not require informed consent. None of the authors on this paper served on the Sun Health IRB and the granting bodies that provided funding for this study did not require any type of ethics review.

### Neuropsychological Tests

#### Rey Auditory Verbal Learning Test [14]

A list of 15 words is read aloud to the individual after which they are asked to recall as many words as possible in any order. This is done 5 times. After the fifth trial, a new 15-word list is read aloud to the individual after which they are asked to recall as many words as possible in any order. They are then asked to recall the words they remember from the list that was read to them 5 times. After a 20 minute delay, they are again asked to recall words from the list that was read 5 times.

#### WMS-R Logical Memory [15]

A short fictional story is read to the individual after which they are asked to repeat as much of the story as they can remember. After a 20 minute delay, they are asked to recall the story again.

#### Trails A [16]

The individual is instructed to draw a line that connects circled numbers in consecutive order.

#### Trails B [16]

The individual is asked to draw a line that connects circled numbers and circled letters in consecutive order while alternating between numbers and letters (1 - A - 2 - B - 3 - C, etc).

#### Controlled Oral Word Association Test [14]

Individuals are given one minute to verbally produce as many words as they can that begin a given letter. One minute per word is given.

#### Animal Fluency [14]

Individuals are given one minute to verbally produce as many names of animals as they can.

#### Stroop Color/Word [17]

The individual is presented with 5 columns of the words "blue", "red", and "green" presented in random order. The words are printed in an ink that is incongruent with the actual word itself (ie, the word "blue" is printed in red ink). The individual is then asked to identify the color of the ink the word is printed in. There is a 45-second time limit in which the individual must give as many correct responses as possible.

#### Judgment of Line Orientation [18]

Individuals are asked to match a set of two lines set at varying angles and lengths to a reference of lines placed below each stimulus card for each trial.

### The Alzheimer's Questionnaire (AQ)

The Alzheimer's Questionnaire (AQ) is a 21-item, informant-based dementia assessment designed for ease of use in a primary care setting. AQ items are divided into five domains including Memory, Orientation, Functional Ability, Visuospatial Ability, and Language. Items are posed in a yes/no format with the sum of 'yes' items equaling the total AQ score (0-27). Six items known to be predictive of a clinical AD diagnosis are weighted more heavily in the total score by being worth two points rather than one.

### Statistical Analysis

Data from the individual AQ items were first analyzed using the Chi-square statistic to determine if there were significant differences in positive response frequencies between aMCI and CN individuals for each item. Multiple forward stepwise logistic regression was carried out to determine the predictive ability of individual AQ items while adjusting for the effects of age and education. For this analysis, clinical status (aMCI) was the outcome and the individual AQ items were entered as predictors. Criteria for retaining predictor variables was set to alpha < .05. Nagelkerke's R^2 ^was used to determine the amount of variance accounted for by the logistic model.

Systat 13.0 was used to carry out all analyses.

## Results

Demographic characteristics of the study sample are displayed in Table 1. The CN group was older and slightly more educated than the aMCI group. Males and females had relatively equal representation across groups. Chi-square analysis showed significant differences in response frequencies for all but two AQ items (Table 2). Results from the multiple forward stepwise logistic regression analysis, which adjusted for age and education, are displayed in Table 3; only the AQ items that were included in the stepwise model are shown. This model yielded four AQ items as strong predictors of aMCI, which are listed in Table 3 with their associated odds ratios (OR), 95% confidence intervals, and *p*-values. The resulting stepwise logistic model yielded a Nagelkerke R^2 ^of 0.71 indicating that a large proportion of the variance between aMCI and CN individuals was accounted for by the four AQ items.

**Table 1:** Demographic Characteristics

	*CN*	*aMCI*	*Total*
N	51	47	98
Male (%)	43	57	50
Female (%)	57	43	50
Age	78.59 (6.72)	74.36 (7.19)	76.56 (7.23)
Education	15.04 (3.03)	14.43 (2.51)	14.74 (2.79)
MMSE	28.47 (1.27)	26.89 (1.90)	27.71 (1.78)

Mean (sd)

**Table 2:** Frequency of Informant-Reported Cognitive Symptoms for aMCI and CN Groups

*Item*	*χ^2^*	*p-value*	*Yes - aMCI*	*Yes - CN*
Does the patient have memory loss?	25.99	< .0001	46/47	27/51
If so, is their memory worse than a few years ago?	6.30	.01	33/47	23/51
Does the patient repeat questions or statements or stories in the same day?	37.60	< .0001	33/47	5/51
Have you had to take over tracking events or appointments, or does the patient forget appointments?	21.77	< .0001	30/47	9/51
Does the patient misplace items more than once a month, or does the patient misplace objects so that he/she cannot find them?	11.95	.005	33/47	18/51
Does the patient suspect others of moving, hiding, or stealing items when he/she cannot find them?	3.53	.06	7/47	2/51
Does the patient frequently have trouble knowing the day, date, month, year, and time; or does the patient reference a newspaper or calendar for the date more than once a day?	17.79	< .0001	18/47	2/51
Does the patient become disoriented in unfamiliar places?	17.78	< .0001	24/47	6/51
Does the patient become more confused when travelling outside the home?	9.87	.0017	21/47	8/51
Excluding physical limitations, does the patient have trouble handling money (tips, calculating change)?	5.72	.02*	5/47	0/51
Excluding physical limitations, does the patient have trouble paying bills or doing finances; or are family members taking over because of concerns about ability?	19.91	< .0001	21/47	3/51
Does the patient have trouble remembering to take medications or tracking medications taken?	16.76	< .0001	19/47	3/51
Is the patient having difficulty driving; or are you concerned about the patient's driving; or has the patient stopped driving for reasons other than physical limitations?	0.50	.48	11/47	9/51
Is the patient having trouble using appliances?	4.31	.05*	6/47	1/51
Excluding physical limitations, is the patient having difficulty in completing home repair or housekeeping tasks?	9.16	.003	10/47	1/51
Is the patient getting lost in familiar surroundings?	4.31	.04	6/47	1/51
Does the patient have a decreased sense of direction?	19.99	< .0001	24/47	5/51
Does the patient have trouble finding words other than names?	6.81	.009	24/47	13/51
Does the patient confuse names of family members or friends?	15.94	< .0001	20/47	4/51
Does the patient have difficulty recognizing people familiar to him/her?	6.94	.009	6/47	0/51

* Fisher's exact test p-value was used due to expected cell counts less than 5

**Table 3:** Multiple Forward Stepwise Logistic Regression Analysis of AQ Items

*AQ Item*	*Odds Ratio*	*95% CI*	*p-value*
Does the patient repeat questions or statements or stories in the same day?	13.12	(3.02, 57.66)	0.001
Does the patient frequently have trouble knowing the day, date, month, year, and time; or does the patient reference a newspaper or calendar for the date more than once a day?	17.97	(2.63, 122.77)	0.003
Excluding physical limitations, does the patient have trouble paying bills or doing finances; or are family members taking over because of concerns about ability?	11.60	(2.10, 63.99)	0.005
Does the patient have a decreased sense of direction?	5.84	(1.09, 32.30)	0.04

In order to more accurately characterize the clinical validity of these findings, a second non-stepwise logistic regression analysis was carried out which used only the four significant AQ items while correcting for age and education. This model yielded sensitivity of 80.30 (67.00, 89.53) and specificity of 81.80 (69.67, 90.37) with an area under the curve (AUC) value of 0.94 (0.89, 0.99).

## Discussion and Conclusions

The results of the study indicate that the four informant-reported items listed immediately above are highly predictive of aMCI. These items are memory-related, and also suggest some degree of impairment in higher-level functional abilities. The use of informant-supplied information is a widely-used and highly valid method of assessing an individual's cognitive and functional abilities [5,7]. Relative to other informant-based instruments [19-22] the AQ takes substantially less time to administer [12], a fact of importance to clinicians with very limited time [23].

For clinicians who see patients with subjective memory complaints, accurate identification of those who need further evaluation is critical to cost containment and resource management. A significant proportion of older adults present with subjective memory complaints [24,25], and these complaints can precede the onset of clinical AD [26]. The large and growing number of older adults underscores the importance of utilizing brief and accurate screening measures. Additionally, as new therapies for AD transition from being symptomatic to disease-modifying, identifying individuals who are at-risk or are in the earliest stages of the disease will be crucial in determining and improving disease outcome [1].

There are some limitations to this study. The first is that the confidence intervals for the odds ratios of the statistically significant AQ items were relatively wide, indicating decreased statistical power. Although the sample was large enough to yield robust odds ratios for the four AQ items, a larger sample size might provide a more accurate estimate of effect size. In addition, the R^2 ^value may not truly represent the amount of variance accounted for by the model. The reason for this is that R^2 ^values in logistic models are approximations of linear-based R^2 ^measures and are not fully equivalent. In addition, R^2 ^measures used in logistic models are prone to bias when used with small sample sizes and may result in an inflated estimate of the amount of variance accounted for [27]. Another limitation is that the AQ itself requires the use of an informant. In some cases, a patient may come to a physician's office alone or they may not have a reliable informant available to do the assessment. Although several patient-based cognitive assessments, such as the Mini Mental State Exam [28], can be used, they are subject to confounding factors such as cultural effects and low education [29-31]. Finally, the study sample was homogenous with respect to ethnicity, as all subjects were Caucasian, so it is unclear whether these results are applicable to an ethnically diverse population.

In addition, the ability of other widely-used informant-based instruments to accurately identify clinical aMCI has not been established. The validity and accuracy of the AD8 has been established in clinical AD and in individuals with a CDR global rating 0.5 which is considered "very mild dementia" [32]. It is important to note that this categorization (CDR = 0.5) does not necessarily equate to a clinical diagnosis of aMCI so it is uncertain whether the AD8 can accurately identify clinically-defined aMCI cases. In addition, a recent study demonstrated that the IQCODE does not have high sensitivity in the detection of aMCI [33]. As mentioned earlier, a previous pilot study of the AQ demonstrated high sensitivity and specificity for aMCI when compared to cognitively normal individuals. The results of the current study showed that four statistically significant AQ items accounted for large proportion of the variance between aMCI and CN individuals and also yielded high sensitivity and specificity in differentiating the two groups. Overall, the results of this study indicate that certain AQ items can differentiate individuals with aMCI from those experiencing age-associated changes in memory and cognition. As assessed by the AQ, difficulties with orientation to time, repetition of questions and statements, difficulties in managing finances, and visuospatial disorientation were all significant predictors of aMCI as diagnosed by an expert in memory disorders.

Given that memory complaints are commonly reported by elderly patients and their family members [7], a means to quickly and accurately identify individuals who may be in the early stages of AD and in need of further evaluation is critical to not only cost containment and resource management, but also to earlier diagnosis in order to improve disease outcome. These data indicate that problems with orientation to time, repeating statements and questions, difficulty managing finances, and trouble with visusospatial orientation may accompany memory deficits in aMCI. From a clinical standpoint, these findings are important as it will allow clinicians to more easily and accurately determine which individuals require further assessment of cognitive problems.

## List of abbreviations

AD: Alzheimer's disease; AQ: Alzheimer's Questionnaire; aMCI: amnestic mild cognitive impairment; CN: cognitively normal; OR: odds ratio.

## Competing interests

None of the authors have competing interests to report. The AQ is copyrighted by Banner Health and is freely available by submitting a request to the authors via e-mail.

## Authors' contributions

All authors read and approved the final version of the manuscript. MM developed the study hypothesis, conducted the statistical analysis, drafted the manuscript, and administered the AQ. KD administered the AQ. CB aided in drafting the manuscript and administered the AQ. SJ aided in drafting the manuscript and administered the AQ. MS developed the research hypothesis, administered the AQ, and aided in drafting the manuscript.

## Pre-publication history

The pre-publication history for this paper can be accessed here:

http://www.biomedcentral.com/1471-2318/12/3/prepub
